# A History of Concussion Affects Relevancy-Based Modulation of Cortical Responses to Tactile Stimuli

**DOI:** 10.3389/fnint.2020.00033

**Published:** 2020-07-03

**Authors:** Meaghan S. Adams, Ewa Niechwiej-Szwedo, William E. McIlroy, William R. Staines

**Affiliations:** Department of Kinesiology, University of Waterloo, Waterloo, ON, Canada

**Keywords:** task-relevance, electroencephalography, somatosensory ERP, somatosensory processing, sensory gating, concussion

## Abstract

Modulating cortical excitability based on a stimulus’ relevance to the task at hand is a component of sensory gating, and serves to protect higher cortical centers from being overwhelmed with irrelevant information (McIlroy et al., 2003; Kumar et al., 2005; Wasaka et al., 2005). This study examined relevancy-based modulation of cortical excitability, and corresponding behavioral responses, in the face of distracting stimuli in participants with and without a history of concussion (mean age 22 ± 3 SD years; most recent concussion 39.1 ± 30 SD months). Participants were required to make a scaled motor response to the amplitudes of visual and tactile stimuli presented individually or concurrently. Task relevance was manipulated, and stimuli were occasionally presented with irrelevant distractors. Electroencephalography (EEG) and task accuracy data were collected from participants with and without a history of concussion. The somatosensory-evoked N70 event-related potential (ERP) was significantly modulated by task relevance in the control group but not in those with a history of concussion, and there was a significantly greater cost to task accuracy in the concussion history group when relevant stimuli were presented with an irrelevant distractor. This study demonstrated that relevancy-based modulation of electrophysiological responses and behavioral correlates of sensory gating differ in people with and without a history of concussion, even after patients were symptom-free and considered recovered from their injuries.

## Introduction

We are surrounded by multiple competing stimuli at all times during daily life, but not all stimuli need to elicit equivalent cortical responses. Sensory gating is the process by which the transmission of sensory information from the periphery to the cortex can be modulated to prevent overwhelming higher cortical centers with irrelevant information (McIlroy et al., 2003; Kumar et al., 2005; Wasaka et al., 2005). The relevance of a stimulus to the task at hand is a contributor to how the stimulus is processed, with more relevant stimuli eliciting more robust cortical responses. Previous work by our lab demonstrated that the somatosensory-evoked N70 potential is enhanced when the evoking stimulus is task-relevant and attenuated when the evoking stimulus is irrelevant during a sensory grading task (Adams et al., 2017, 2019). When the ability to downregulate cortical excitability to irrelevant distractor stimuli was disrupted using cTBS applied to the prefrontal cortex, these distractors exerted a greater behavioral cost during the sensory grading task (Adams et al., 2019).

Brain damage affecting the prefrontal cortex has disruptive effects on sensory gating processes (Knight et al., 1989, 1999; Yamaguchi and Knight, 1990) and patients with these diagnoses demonstrate behavioral changes, including difficulty using contextual information to complete tasks, increased distractibility, and decreased attention capacity (Knight et al., 1989; Yamaguchi and Knight, 1990; Fogelson et al., 2009). After traumatic brain injuries, patients frequently report difficulties sustaining attention, particularly in complex environments with multiple competing sensory stimuli, such as grocery stores or noisy rooms (Arciniegas et al., 1999; Halterman et al., 2006). We hypothesized that these concussion sequelae may be related to impairments in the cortical processes related to gating irrelevant stimuli out of the processing stream. Since the tactile-evoked N70 potential had been shown to have relevancy effects in two separate groups of healthy young adults, and these relevancy effects could be linked to performance accuracy on our sensory grading task, we sought to examine relevancy-based sensory gating in a group of people with a history of concussion. Our objective was to explore differences in cortical and behavioral responses in a group of people who had recovered from concussions.

We chose a group who had recovered from a concussion because there is growing evidence that concussions leave lasting effects on patients, even after symptoms have resolved. Compared to controls with no history of concussion, patients who have recovered from concussion have deficits in visuomotor control, decision-making, and dynamic stability (Baker and Cinelli, 2014); longer reaction times on a complex visuomotor mapping task (Hurtubise et al., 2016); and white matter abnormalities and resting-state connectivity changes on fMRI (Manning et al., 2017). Other studies using electroencephalography (EEG) in this population have shown that patients with a history of concussion, even when asymptomatic, display decreased amplitudes of specific event-related potentials (ERPs) on EEG (De Beaumont et al., 2007, 2012; Thériault et al., 2009, 2011; Baillargeon et al., 2012). ERPs have also been used to examine changes in working memory, attention, and motor function after concussion (De Beaumont et al., 2007, 2012; Thériault et al., 2009, 2011; Baillargeon et al., 2012; Gosselin et al., 2012), and there is evidence that some of these changes persist even after symptoms have resolved and patients have resumed normal activities.

The present study was designed to investigate how a history of concussion affected the tactile-evoked N70 ERP, which we have shown to be a cortical correlate of task relevance and distractibility in two separate groups of participants (Adams et al., 2017, 2019). The first hypothesis of the present experiment was that relevancy-based sensory gating would be impaired in participants with a history of concussion, resulting in less suppression of N70 cortical responses to task-irrelevant stimuli. The second hypothesis was that the presentation of unattended distractor stimuli would negatively affect the accuracy of the visual grading task in those with a history of concussion, due to the hypothesized disruption in early relevancy-based gating of tactile stimuli.

## Materials and Methods

### Participants

EEG and behavioral data were collected from a total of 27 volunteers: 14 with a history of concussion (eight female, six male, aged 18–31), and 13 with no history of concussion (eight female, five male, aged 19–28). All participants in the control group had no history of a diagnosed or suspected concussion. Of the 14 participants in the concussion history group, seven had been previously diagnosed with one concussion, three had been diagnosed with two, three had been diagnosed with three, and one had been diagnosed with nine concussions in the past. All 14 of these participants were considered fully recovered and symptom-free by current clinical criteria and were medically cleared to return to full participation in school or work, activities of daily living, and sporting activities. No restrictions were placed on the maximum number of concussions, time since the most recent concussion, recovery time, or age at the time of injury for those in the concussion group, ensuring that the participant group reflected the heterogeneity of the wider population of those who have recovered from concussion and consistent with other published studies (Thériault et al., 2009, 2011; Dalecki et al., 2016; Hurtubise et al., 2016); see Table 1 for participant characteristics. Control participant data have been examined and published previously (Adams et al., 2017). Participants had no history of substance abuse, psychoactive drug treatment, or neurological disease or impairment, other than a concussion(s) for those in the concussion history group. All experimental procedures were approved by the University of Waterloo’s Office of Research Ethics, and all participants provided written informed consent to participate.

**Table 1:** Demographic information for control and concussion history participants.

Group	Number of particpants	Age (years) ± SD	Number of concussions ± SD	Time since most recent injury (months) ± SD	Length of recovery from most recent injury (months) ± SD
Control	13	21.9 ± 2.7	0	-	-
Concussion history	14	22.1 ± 3.9	2.3 ± 2.2	39.1 ± 30.2	4.0 ± 4.8

*The number of concussions refers to self-report of medically diagnosed concussions. Values are expressed as means ± standard deviation*.

### Experimental Design

The experimental task required participants to approximate the amplitude of discrete visual and tactile stimuli by applying a graded motor response to a pressure-sensitive bulb. The stimuli were presented either in isolation, as unimodal tactile (T) or visual (V) stimuli, or simultaneously, as crossmodal visual and tactile stimuli (VT). A single trial consisted of tactile, visual, or dual stimulus presentation. Experimental blocks lasted for approximately three and a half minutes and contained 54 stimuli each presented for 500 ms, with 2.5 s between trials. The experimental design consisted of ten blocks of trials divided among two attention manipulations, five blocks per manipulation, presented in random order. Participants were required to attend, and produce a force-graded response, to approximate the amplitude of tactile stimuli (presented as unimodal or crossmodal) during the tactile grading blocks, and visual stimuli (presented as unimodal or crossmodal) during the visual grading blocks. There were some trials during each block where participants did not make a response (i.e., when participants were asked to attend and respond to tactile stimuli, they made no response when unimodal visual stimuli were presented).

### Experimental Paradigm

**Figure 1:**
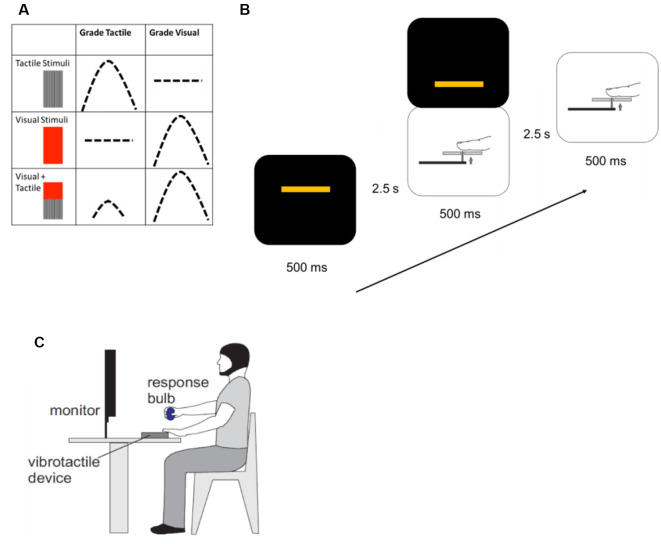
Methods. **(A)** Each experimental trial consisted of a unimodal tactile stimulus, a unimodal visual stimulus, or simultaneously-presented visual and tactile stimuli. After each trial was presented, participants made a force-graded response to approximate the amplitude of the target stimulus. During blocks when the instruction was to grade tactile stimuli, participants would respond to either unimodal or crossmodal tactile stimuli. Similarly, during blocks when the instruction was to grade visual stimuli, participants would approximate the amplitude of unimodal or crossmodal visual stimuli. Instructions to participants were varied randomly for each block. **(B)** One experimental block contained 54 trials. Unimodal or crossmodal stimuli were presented for a total of 500 ms, with 2.5 s between stimuli to allow participants to respond. Stimuli were presented in random order. **(C)** Participants were seated, with a pressure-sensitive bulb in their right hand, their left hands resting on a vibrotactile delivery device, and maintaining visual fixation on a computer screen for the presentation of visual stimuli.

Each participant was seated comfortably for the duration of the experiment. They fixed their gaze on a computer screen for all blocks and rested the palmar surface of the second digit of the left hand on a device which delivered vibrotactile stimuli. Participants judged the amplitude of the stimulus type they were instructed to respond to, or track, for that block: either tactile alone or visual alone; and made a graded motor response by squeezing a pressure-sensitive rubber bulb with their right hand. When responding to tactile stimuli, participants were asked to apply enough force to the pressure-sensitive bulb to approximate the vibration amplitude of each tactile stimulus presented. They were asked to do this each time a tactile stimulus was presented, whether it was presented alone or in combination with a visual one. The visual condition was similar, with participants applying force to the bulb to correspond to the height of a bar appearing on the computer screen, regardless of whether or not a tactile stimulus accompanied it (Figure 1).

The experimental trials were preceded by a training session, consisting of 40 training trials and lasting approximately 5 min. In each training trial, two bars were presented on the computer screen: a blue bar, controlled by the participant squeezing the pressure bulb, and a yellow one which varied randomly in height. The aim was for participants to raise the blue bar to the height of the yellow bar by applying a graded force to the pressure bulb. The blue bar provided visual feedback to teach participants how to use force to grade the visual stimuli. At the same time, the amplitude of the vibrotactile stimulus applied to the subject’s finger varied proportionally to match the force applied to the bulb. In this way, the training program connected the visual and vibrotactile stimuli through the means of the force applied to the pressure-sensitive bulb. During experimental trials, the blue response bar was absent, depriving participants of feedback about the accuracy of their grading performance, and the amplitude of the vibrotactile stimuli varied independently of the visual stimuli.

### Stimuli

The target visual stimulus was a yellow bar (6 cm wide) which appeared in the center of a black box presented on a black computer screen. The bar was visible for 500 ms and appeared at randomized heights within the box. Tactile stimuli were delivered to the second digit of the left hand using a custom-made vibrotactile device. These stimuli were created by the conversion of digitally-generated waveforms to analogue signals (DAQCard 6024E, National Instruments, Austin, TX, USA) and amplifying the signal (Bryston 2BLP, Peterborough, ON, Canada) using a custom program written in LabVIEW (version 8.5; National Instruments). Variations in the amplitude of the voltage driving the vibrotactile device resulted in proportional changes in the amplitude of the tactile stimulus applied to the finger. The amplitude of each vibration was constant within a trial and varied randomly between trials. The average stimulus amplitude across all trials which included a tactile stimulus did not differ between the experimental conditions, and the frequency of the vibration was held constant at 25 Hz. To prevent the auditory perception of the vibrotactile stimuli, participants wore earbud headphones during the experiment which delivered white noise throughout the training and experimental tasks (White Noise Ambience Lite, Logicworks version 2.70, Apple App Store).

### Data Acquisition and Recording Parameters

Behavioral data were recorded using a custom program written in LabVIEW (version 8.5, National Instruments, Austin, TX, USA). Participants applied force to the pressure-sensitive bulb that caused air to move through a rubber tube in a closed system, leading to a pressure change that was measured by a pressure sensor and converted to a voltage. There was a linear relationship between the pressure measurement and the voltage produced. EEG data were recorded from 32 electrode sites (32 channel Quik-Cap, Neuroscan, Compumedics, NC, USA) following the international 10–20 system for electrode placement and referenced to the linked mastoids. Impedance was maintained less than 5 kΩ. EEG data were collected with a DC—100 Hz filter and digitized at 500 Hz (Neuroscan 4.5, SynAmps2, Compumedics, NC, USA). Data were then saved for subsequent analysis.

### Data Analysis

#### EEG Analysis

Analysis of the EEG data began with epoching, followed by baseline correction to the pre-stimulus interval and the application of a 0.1–50 Hz bandpass filter. Epochs were 600 ms in length, beginning 100 ms before stimulus onset, and epochs contaminated by blinks, muscle contractions, or eye movements were eliminated by visual inspection before averaging. Between 90 and 108 trials per participant were collected for each stimulus type, and after contaminated trials were eliminated, the final trace for each experimental condition consisted of an average of 62 artifact-free epochs for individuals in the control group, and 83 artifact-free epochs for those in the concussion history group.

Mean ERP amplitudes and latencies were computed for each subject within specific time windows centered around the post-stimulus latencies of early somatosensory and visual ERP components: somatosensory—P50 (45–75 ms), N70 (60–80 ms), P100 (80–120 ms), N140 (125–175 ms); visual—P1 (125–175 ms), N1 (180–220 ms) and P2 (225–285 ms). For all ERP analysis, potentials were calculated as peak-to-peak amplitudes between the peak of interest and the preceding potential of opposite polarity, except for the P50 amplitude which was calculated relative to the baseline. A clearly defined peak was necessary for inclusion. Separate three-way mixed-model ANOVAs were carried out on the amplitudes and latencies of each potential to make between-group comparisons, with attention instruction (T, V), the stimulus presented (T, V, VT), as within-subject factors, and group (control, concussion history) as the between-subject factor. Data sets were tested for normality to validate the use of parametric tests, and transformed when necessary to uphold the assumptions of the ANOVA model. Since N70 amplitudes are modulated by attention in the control group (Adams et al., 2017), pre-planned contrasts were conducted on the N70 potential in the post-concussion group. Specifically, contrasts tested the hypotheses that the modulation of N70 amplitude by task-relevance which was seen in the control group would not be replicated in the group with concussion history and that the presentation of a task-irrelevant visual distractor would significantly decrease the tactile-evoked N70 amplitude in the concussion history group.

#### Behavioral Analysis

Behavioral data were analyzed by comparing the amplitude of the target stimulus to the amplitude of the response created by the participant squeezing the pressure-sensitive bulb. The response was compared to the amplitude of the target stimulus to calculate a percentage of the ideal response. Since it was hypothesized that presenting a distracting stimulus would impair accuracy when compared with the undistracted condition, a cost score was calculated. The cost score was the percent of ideal response made during the distracted condition divided by the percent of ideal response from the undistracted condition and multiplying by 100. This was then subtracted from a potential maximal score of 100 to obtain the cost of presenting the distractor. This was done for both the control and concussion history groups, and *t*-tests were used to compare how a history of concussion affected the cost of a distractor on grading in each modality.

## Results

### Event-Related Potentials

#### Tactile ERPs (P50, N70, P100, N140; Table 2)

**Figure 2:**
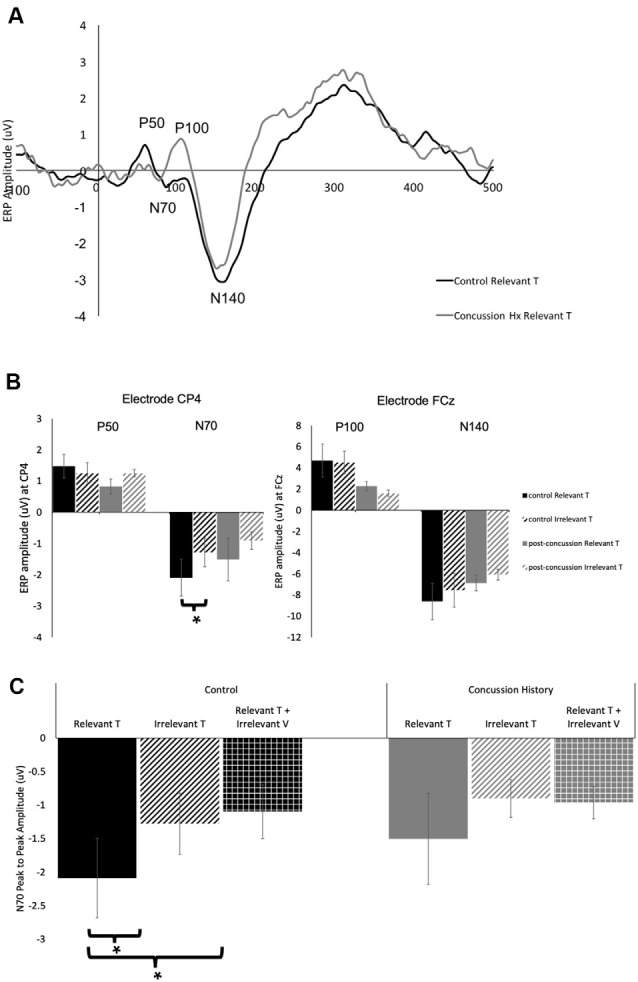
Tactile-evoked event-related potential (ERPs). **(A)** Grand average waveform (*n* = 14), generated in response to the presentation of task-relevant tactile stimuli. ERP components of interest are labeled for electrode CP4. The black trace was generated in the control group and the gray trace was generated from the group with a history of concussion. **(B)** Peak-to-peak tactile-evoked amplitudes in response to task-relevant (solid bars) or task-irrelevant (striped bars) tactile stimuli. P50 and N70 amplitudes were measured at electrode CP4, P100 and N140 amplitudes were measured at FCz. Data collected from the control group is shown in black, and from the concussion history group in gray. N70 amplitudes to tactile stimuli were significantly higher in the control group when tactile stimuli were task-relevant as compared to when they were not. There was no difference in N70 amplitude in the group with a history of concussion, and no significant differences in P50, P100, or N140 amplitude between groups (*indicates significant to *p* < 0.05; error bars indicate a standard error). **(C)** Peak-to-peak N70 amplitude from electrode CP4 to tactile stimuli when the stimuli were task-relevant (solid bars) when they were irrelevant (striped bars), and when they were presented with a simultaneous irrelevant distractor (hatched bars). Data collected from the control group are shown in black, and from the concussion history group in gray. In the control group, the N70 was significantly attenuated when tactile stimuli were task-irrelevant as well as when they were presented with simultaneous distractors (*indicates *p* < 0.05; error bars indicate a standard error). There were no differences between conditions in the concussion history group.

Figure 2A shows grand average traces of tactile ERPs at electrode CP4. All participants in both the control and concussion history groups demonstrated P50 potentials, however, P50 peaks were not elicited in two attention conditions for one individual in the concussion history group.

**Table 2:** Average amplitudes and standard error values for all tactile-evoked event-related potentials (ERPs) at electrodes CP4 and FCz.

		Control								Concussion							
		Relevant T		Irrelevant T		Relevant T + Irrelevant V		Relevant V + Irrelevant T		Relevant T		Irrelevant T		Relevant T + Irrelevant V		Relevant V + Irrelevant T	
		Ave. Amp. (uV)	SE	Ave. Amp. (uV)	SE	Ave. Amp. (uV)	SE	Ave. Amp. (uV)	SE	Ave. Amp. (uV)	SE	Ave. Amp. (uV)	SE	Ave. Amp. (uV)	SE	Ave. Amp. (uV)	SE
CP4	P50	1.48	0.38	1.25	0.35	1.24	0.31	1.41	0.44	0.83	0.25	1.25	0.12	0.83	0.25	0.61	0.25
	N70	−2.09	0.55	−1.28	0.46	−1.10	0.40	1.26	0.37	−1.50	0.68	−1.02	0.30	−0.96	0.24	−1.50	0.68
FCz	P100	4.69	1.57	4.52	1.06	4.04	1.11	−5.50	1.75	2.27	0.44	1.62	0.30	2.64	0.41	2.01	0.31
	N140	−8.60	1.72	−7.56	1.60	−8.88	1.99	9.92	2.29	−6.86	0.74	−6.07	0.51	−7.42	0.79	−6.40	0.80

*Both control and concussion history groups are represented*.

The amplitude and latency of the P50 potential were calculated from the 11 control and 13 concussion history participants who demonstrated clear P50 components. The P50 was generated by unimodal tactile and visuotactile stimuli, and not observed in response to unimodal visual stimuli. It was maximal at electrode CP4 overlying contralateral somatosensory cortex, and analysis was conducted using the potentials from this electrode. In the control group, the mean P50 latency was 58.4 ± SE 1.1 ms, and in the concussion history group, the P50 potential occurred with a mean latency of 53.2 ± SE 0.4 ms. Three-way mixed ANOVA analysis of P50 latency revealed no significant main or interaction effects on P50 latency. The mixed-model ANOVA analysis of P50 amplitude showed a trend toward a significant main effect of group (*F*_(1,25)_ = 3.58, *p* = 0.07) but no significant main effects of stimulus type or attention as well as no significant interactions between any of the factors (Figure 2B).

EEG tracings demonstrated a clear N70 component in response to unimodal tactile and visual-tactile stimuli in all participants (Figure 2A). The N70 was maximal at CP4, overlying contralateral somatosensory cortex, and statistical analysis was conducted using the potentials from this electrode. The mean N70 latency was 78.7 ± SE 1.1 ms in the control group, and 70.25 ± SE 0.51 ms in the concussion history group. A three-way mixed-model ANOVA conducted on N70 latency revealed a significant interaction between the factors group, attention and stimulus (*F*_(2,24)_ = 3.83, *p* = 0.04). This interaction was tested by conducting two separate two-way ANOVAs on the N70 latency values from each group. In the control group, there was a significant interaction between attention and stimulus type (*F*_(1,11)_ = 7.06, *p* = 0.02), while in the concussion history group the interaction between attention and stimulus type trended toward significance (*F*_(1,13)_ = 3.57, *p* = 0.08) but main effects were not significant. Three-way mixed model ANOVA analysis with N70 amplitude as the dependent variable showed a significant three-way interaction effect between the group, attention, and stimulus type (*F*_(2,68)_ = 3.35, *p* = 0.04). This interaction was explored by running a 2-way ANOVAs separately within each group, including pre-planned contrasts as described earlier. In the group with a history of concussion, there were no significant main effects of attention (*F*_(1,33)_ = 0.01, *p* = 0.95), stimulus type (*F*_(1,33)_ = 0.68, *p* = 0.42), nor was there an interaction (*F*_(1,33)_ = 2.47, *p* = 0.12). N70 amplitudes to tactile stimuli were not significantly different when subjects were responding to tactile stimuli [95% CI (−0.17 μV, −2.88 μV)] than when they were responding to visual [95% CI (−0.43 μV, −1.61 μV); *F*_(1,33)_ = 1.25, *p* = 0.27; Figure 2B]. However, data from the control group found that N70 amplitudes to tactile stimuli were significantly larger when subjects were attending and responding to tactile stimuli [95% CI (−1.02 μV, −3.16 μV)] than when they were attending and responding to visual [95% CI (−0.38 μV, −2.17 μV); *F*_(1,58)_ = 5.32, *p* = 0.02; Figure 2B]. The difference between ERP responses to lone tactile stimuli and tactile stimuli presented with a simultaneous visual distractor was also tested, and there was no significant difference in the concussion history group (*F*_(1,37)_ = 2.78, *p* = 0.10). This was in contrast to the control data, which showed that N70 amplitudes were significantly larger when participants with no history of concussion were presented with unimodal tactile stimuli than when the tactile stimulus was presented with a task-irrelevant visual distractor (*F*_(1,58)_ = 7.31, *p* = 0.009; Figure 2C). Visual inspection of the data suggested that the peak N70 amplitudes to relevant tactile stimuli were considerably different between the concussion and the control groups. This difference was not hypothesized before the start of the present experiment, but it was tested using an independent student’s *t*-test between the peak N70 amplitudes to relevant tactile stimuli in each group. There was no significant difference in peak N70 amplitude to relevant tactile stimuli between the control group (*M* = −2.09, *SD* = 3.87) and the group with concussion history (*M* = −1.50, *SD* = 6.05); *t*_(24)_ = −0.67, *p* = 0.51.

EEG tracings collected from all subjects demonstrated a clear P100 component in response to unimodal tactile and visual-tactile stimuli. It was distributed bilaterally at parietal electrode sites and was maximal at electrode FCz, therefore analysis of P100 was conducted at this electrode. The mean P100 latency was 101.2 ± SE 1.4 ms in the control group, and 105.1 ± SE 0.15 ms in the concussion history group. A three-way mixed-model ANOVA with P100 latency as the dependent variable revealed no significant main effects and no interaction effects between any of the factors on the latency of the P100 potential. Three-way mixed-model ANOVA analysis of P100 amplitude showed a significant main effect of group (*F*_(1,25)_ = 5.50, *p* = 0.03), but no significant main effects of stimulus type or attention. There were also no significant interaction effects. The significant main effect of group was explored by completing a two-way repeated-measures ANOVA on the data from the concussion history group and comparing this with the two-way ANOVA conducted previously using control group data. When each group was examined individually, there was no significant interaction between attention and stimulus type, and no significant main effect of attention. The concussion history group had a trend toward a significant main effect of stimulus type (*F*_(1,39)_ = 3.56, *p* = 0.07) which was not present in the control participants (*F*_(1,59)_ = 0.41, *p* = 0.52), and which may explain why the mixed-model ANOVA showed a significant main effect of group. However, since this did not reach significance with additional testing, it was not considered further (Figure 2B).

The N140 component was also demonstrated by all participants in response to unimodal tactile and visual-tactile stimuli, distributed bilaterally and maximal at FCz. The mean N140 latency was 149.5 ± SE 2.2 ms in the control group, and 156.1 ± SE 0.41 ms in the concussion history group. Three-way mixed model ANOVA analysis of N140 latency revealed a significant main effect of stimulus type (*F*_(1,25)_ = 5.79, *p* = 0.02) and a trend toward a significant main effect of attention (*F*_(1,25)_ = 3.66, *p* = 0.07). The main effect of the group did not reach significance nor did any of the interactions between terms (*p* > 0.05). The significant main effect of stimulus type was explored by conducting separate two-way ANOVA analyses of the N140 latency values from each group. In the control group, there was a significant main effect of stimulus type (*F*_(1,12)_ = 4.80, *p* = 0.05) and a trend toward a significant main effect of attention (*F*_(1,12)_ = 4.21, *p* = 0.06) on the latency of the N140 potential, but no interaction between these terms. In the concussion history group, there were no significant main or interaction effects. The amplitude of the N140 potential was also considered: A three-way mixed-model ANOVA of N140 amplitude showed a significant main effect of stimulus type (*F*_(1,25)_ = 11.37, *p* = 0.002), but no significant main effects of group or attention. There was a trend toward a significant interaction effect between group, attention, and stimulus type (*F*_(1,25)_ = 2.97, *p* = 0.07) but no other interaction effects reached significance. The significant main effect of stimulus type was explored by conducting a two-way repeated-measures ANOVA of the N140 amplitudes collected from the concussion history participants for comparison with the control group statistics published previously (Adams et al., 2017). When each group was examined individually, there was no significant interaction between attention and stimulus type in either group and a trend toward a significant main effect of attention in both the control group (*F*_(1,60)_ = 3.60, *p* = 0.06) and the group with a history of concussion (*F*_(1,39)_ = 2.96, *p* = 0.09). There was also a trend toward a significant effect of stimulus type in the group with a history of concussion (*F*_(1,39)_ = 3.33, *p* = 0.08) but not in the control group; since it did not reach statistical significance in either group, the main effect of stimulus type which was shown in the mixed-model ANOVA was not considered further (Figure 2B).

### Visual ERPs (P1, N1, P2; Table 3)

**Figure 3:**
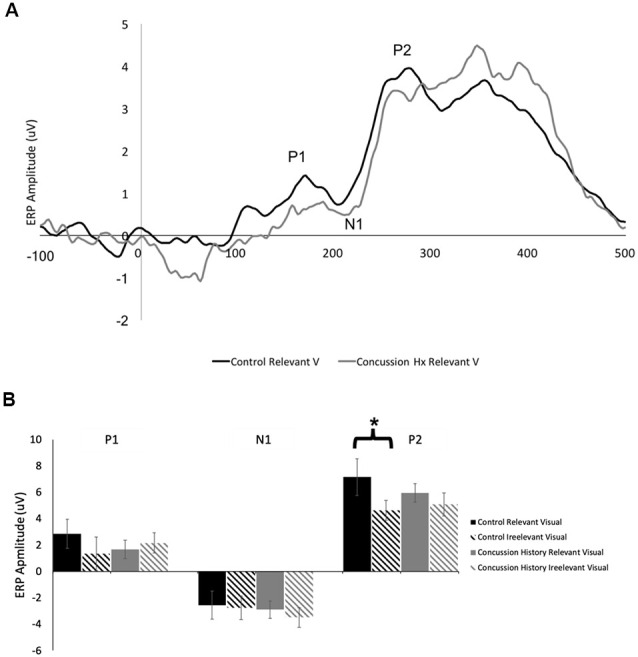
Visually-evoked ERPs. **(A)** Grand average waveform (*n* = 13, generated in response to the presentation of lone visual stimuli. ERP components of interest are labeled for electrode Pz. The black line denotes data generated from the control group, and the gray line was generated from those with a history of concussion. **(B)** Peak-to-peak amplitudes of visually-evoked ERPs at electrode Pz, when visual stimuli were task-relevant (solid bars) and when they were irrelevant (striped bars). Data from control participants is shown in black, and data from those with a history of concussion is shown in gray. In the control group, P2 was significantly attenuated when the evoking visual stimuli were task-irrelevant (*indicates *p* < 0.05; error bars indicate a standard error). There were no differences between conditions in the concussion history group.

Figure 3A shows a grand average trace of the ERPs generated in response to visual stimuli (unimodal visual and visual-tactile) when subjects directed attention toward and away from visual input. Eleven of 13 subjects in the control group and all 14 in the concussion history group demonstrated three clear ERP components in response to visual stimuli, labeled P1, N1, and P2. All were maximal at electrode Pz, distributed bilaterally, and not observed in response to tactile stimuli. Three-way mixed model ANOVA analysis of latency revealed no significant main effects of group, attention, or stimulus type; there were also no interactions between the three factors or between group and attention or group and stimulus type. Mixed model ANOVAs performed for ERP amplitudes showed no significant interaction effects between terms on the amplitude of the visually-evoked ERPs. Main effects of group, stimulus type, or attention also did not reach significance (Figure 3B), however, the mixed-model ANOVA examining the amplitudes of the P2 potential showed a trend toward a significant main effect of stimulus type (*F*_(1,24)_ = 4.05, *p* = 0.06). Since relevancy-based modulation of P2 potentials were not consistently observed in previous work using this paradigm, we did not formulate specific hypotheses about this potential in the present experiment. Therefore, pre-planned contrasts were not conducted.

**Table 3:** Average amplitudes and standard error values for all visually-evoked ERPs at electrode Pz.

		Control								Concussion							
		Relevant T		Irrelevant T		Relevant T + Irrelevant V		Relevant V + Irrelevant T		Relevant T		Irrelevant T		Relevant T + Irrelevant V		Relevant V + Irrelevant T	
		Ave. Amp. (uV)	SE	Ave. Amp. (uV)	SE	Ave. Amp. (uV)	SE	Ave. Amp. (uV)	SE	Ave. Amp. (uV)	SE	Ave. Amp. (uV)	SE	Ave. Amp. (uV)	SE	Ave. Amp. (uV)	SE
Pz	P1	2.84	1.11	1.33	1.26	2.00	0.97	1.11	0.89	1.66	0.69	2.16	0.76	1.63	0.82	2.10	0.76
	N1	−2.56	1.07	−2.75	0.89	2.96	0.52	3.07	0.56	−2.89	0.66	−3.49	0.75	−3.72	0.01	−3.72	0.83
	P2	7.15	1.38	4.63	0.76	7.41	1.80	6.78	1.38	5.95	0.70	5.08	0.87	10.57	3.26	7.08	1.04

*Both control and concussion history groups are represented*.

## Behavioral Performance

**Figure 4:**
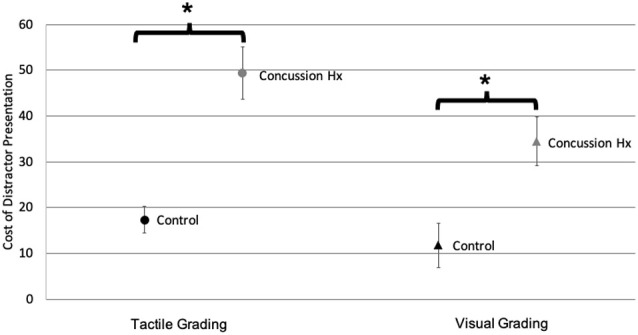
Sensory grading task accuracy. Accuracy cost when target stimuli are presented with simultaneous distractors, for both tactile (circles) and visual (triangles) grading conditions. Black markers represent the control group; gray markers represent the concussion history group. There was a significant increase in distractor cost during both grading conditions in the group with a history of concussion, as compared to controls (Error bars denote standard deviation). *p < 0.05.

The amplitude of the target stimulus was compared to the amplitude of the response created by the participant squeezing the pressure-sensitive bulb to calculate a percentage of the ideal response. These responses to unimodal tactile and visual stimuli were compared between participant groups using independent student’s *t*-tests. There was no significant difference (*t*_(25)_ = −0.53, *p* = 0.67) in the mean percent ideal response to unimodal tactile stimuli between the control (*M* = 156.8, *SD* = 114.4) and concussion history groups (*M* = 173.4, *SD* = 81.9). There was also no significant difference (*t*_(25)_ = −0.12, *p* = 0.9) in the mean percent ideal response to unimodal visual stimuli between the control (*M* = 206.7, *SD* = 86.0) and concussion history groups (*M* = 210.7, *SD* = 80.6). To improve the interpretation of these behavioral data, a cost score was also calculated to represent the change in accuracy caused by the presentation of a simultaneous distractor stimulus, and independent student’s *t*-tests were also conducted within each sensory modality to test the change in accuracy caused by a distractor in the control group as compared to the group with a history of concussion (Figure 4). For tactile grading, there was a significantly greater cost of a visual distractor on task accuracy (*t*_(19)_ = −5.01, *p* < 0.0001) in the concussion history group (*M* = 49.32, *SD* = 21.27) as compared to the control group (*M* = 17.37, *SD* = 10.42). Similarly for visual grading, there was a significantly greater cost of a tactile distractor on task accuracy (*t*_(25)_ = −3.15, *p* = 0.02) in the concussion history group (*M* = 34.4, *SD* = 19.40) as compared to the control group (*M* = 11.81, *SD* = 17.29).

## Discussion

The present study demonstrated that individuals with a history of concussion did not experience significant modulation of the tactile-evoked N70 ERP as the evoking tactile stimuli varied in task relevance, in direct contrast to a group who had never been diagnosed with a concussion. It was expected that a disruption in the modulation of cortical excitability by stimulus relevance would affect behavioral outcomes, and the results in both the visual and tactile modalities showed a significantly greater behavioral cost of presenting a distractor stimulus in the group with a history of concussion than in the control group.

### Effect of Concussion on Relevancy-Based Gating

The first hypothesis of the present study, that a history of concussion would impair relevancy-based sensory gating, was supported by the results of the experiment. In the concussion history group, the amplitude of the tactile-evoked N70 was not significantly different when the evoking stimulus modality varied in task relevance. This is in contrast to the significantly larger and later latency N70 potential seen in the control group when the evoking stimuli were relevant to the sensory grading task. The effects of amplitude and latency here are likely related: a larger-amplitude waveform would take longer to reach its peak and return to baseline. The loss of N70 modulation by task relevance in the concussion history group appears to be related to less peak-to-peak N70 enhancement in the task-relevant condition (95% confidence interval near zero) and the increased variability of ERP amplitudes in this group, in addition to less attenuation in the task-irrelevant condition.

A similar pattern has been demonstrated in patients with prefrontal cortical lesions, who show less attenuation of auditory ERP amplitude in response to distractor stimuli, suggesting a failure in inhibitory control over the processing of irrelevant stimuli and implicating the PFC in the modulation of cortical responses to sensory stimuli based on their relevance to an experimental task (Knight et al., 1999). Patients with PFC lesions have also shown decreased early (125 ms) and late (200–650 ms) cortical responses to visual stimuli (Barceló et al., 2000). This similarity should not be interpreted as causal: the PFC is not, and should not be considered, solely responsible for concussion-related deficits. However, the accumulation of evidence showing that concussion disturbs functions such as sensory gating and working memory, which are understood to be prefrontal-mediated (Knight et al., 1999; Hillary et al., 2010; Bolton and Staines, 2011; Brown et al., 2015), suggests a relationship we do not currently understand between the highly interconnected PFC and concussion injuries. This experiment was not designed to directly assess the role of the PFC in concussion-related symptoms and deficits, but raises questions about the functional implications of the injury.

There is a growing body of literature showing electrophysiological changes in patients after a concussion. Compared to a control group, participants with symptomatic concussion injuries showed decreased N350 and P300 amplitudes, as well as slower reaction times and decreased task accuracy, during a visual working memory task (Gosselin et al., 2012). EEG changes have also been shown in groups who had recovered from a concussion, similar to the population studied in the present experiment (De Beaumont et al., 2012; Gosselin et al., 2012). Most literature has examined changes in the P3 or P300 potential (De Beaumont et al., 2007, 2012), an index of attention and cognitive efficiency (De Beaumont et al., 2012; Gosselin et al., 2012). The P3 ERP is selectively suppressed in amplitude during a visual oddball task in a group of participants who had recovered from a concussion, with the greatest degree of suppression in participants who had sustained a greater number of concussions (De Beaumont et al., 2007). In the same patients, the N2pc potential, related to visuospatial attention, was unaffected by concussion history, confirming that a history of concussion does not generally suppress cortical activity but exerts specific effects only on certain potentials during the performance of particular tasks (De Beaumont et al., 2007). Suppression or loss of modulation of general indices of cortical function is consistent with the loss of modulation of modality-specific ERPs shown in the present experiment. The present study provides further evidence that concussion exerts specific effects on certain event-related potentials, rather than causing an overall suppression of cortical activity, and does so even when symptoms have resolved and patients are considered medically recovered.

### Effect of Distractor Stimuli on Task Accuracy

The second hypothesis of the current experiment was that the presentation of unattended distractor stimuli would negatively affect task accuracy in the visual grading task in the group with a history of concussion. This was based on the expectation that gating based on task-relevance would be disrupted after a concussion, allowing stimuli into the processing stream which were previously gated out of it, and negatively affecting behavior. The results of the present study show that the behavioral cost of presenting a simultaneous distractor was significantly higher in the concussion history group than in the control group in both sensory modalities.

The increased cost to task accuracy in the present experiment may be explained by the impairment in relevancy-based modulation shown in the electrophysiological results. Participants with a history of concussion demonstrated less relevancy-based ERP modulation. It is conceivable that, if stimuli are not modulated by their relevance as effectively, they may be more distracting when presented as irrelevant stimuli. The comparable cost of distractor presentation demonstrated in the present experiment for both sensory grading tasks suggests that stimuli in both sensory modalities were not effectively gated out of the processing stream.

However, the correlation of electrophysiological findings with the results of behavioral tests should be done with caution. Electrophysiology is one of a variety of physiological determinants of behavioral outcomes, and a one-to-one relationship between EEG and behavior should not be assumed. Correlation of electrophysiological findings with the results of behavioral tests is particularly inconsistent in populations after a concussion, and two main theories exist to explain this discrepancy. One theory states that the brain uses its available resources to compensate for damage by differentially recruiting other brain networks or by utilizing alternative cognitive strategies to optimize performance, a concept that is known as a cognitive reserve (Thériault et al., 2011; De Beaumont et al., 2012). If participants can access cognitive resources held in “reserve” or change their cognitive strategy to maintain baseline functional performance (Thériault et al., 2011), it may help to explain how significant ERP waveform changes post-concussion can coexist with baseline-level performance on neuropsychological tests. An alternative explanation for the discrepancy between ERP changes and task performance may be that recovery from concussion is a two-step process. The first step, involving compensatory mechanisms, produces rapid initial recovery of function; this is followed by a second step consisting of prolonged neuronal recovery (Baillargeon et al., 2012). It is during this period of long-term recovery that deficits may be apparent on electrophysiological measures while task performance may appear recovered.

Complex activities, such as those requiring dual-tasking are more sensitive to performance decrements after concussion (Tapper et al., 2017). Task type also contributes to differences between task performance and electrophysiology. Even without a dual-tasking component, the task used in the present experiment showed that grading accuracy suffered when a distractor stimulus was delivered to those with a history of concussion, even though these participants were no longer experiencing concussion symptoms (see Figure 4). This raises the question of how recovery from a concussion should be defined and suggests that relying on symptom resolution (McCrory et al., 2017) is an incomplete metric upon which to base decisions about concussion recovery.

People with symptomatic injuries have been shown to have both decreased performance on working memory tasks as well as decreases in ERPs associated with working memory and attention processes (Gosselin et al., 2012). However, measures of both task performance and cortical function show much more variable results when examined in individuals who have recovered and are asymptomatic at the time of testing, such as the participants in the present experiment. While normal performance has been shown on a visual search oddball task (De Beaumont et al., 2007), other test paradigms have demonstrated deficits on a diverse range of behavioral and neurophysiological outcome measures (Thériault et al., 2009; Slobounov et al., 2012; Baker and Cinelli, 2014; Tapper et al., 2017; Manning et al., 2017), and still, others have shown significant differences in electrophysiology or task performance only when participants were stratified according to the number of concussions they had sustained (Thériault et al., 2011; Hurtubise et al., 2016). The present study adds to the growing body of literature suggesting that resolution of concussion symptoms does not indicate full physiologic recovery. This has substantial implications for clinical management and rehabilitation after a concussion, which currently bases patient care decisions, such as return to school or sport, on a patient’s self-reported symptoms (McCrory et al., 2017), and is a topic which requires substantial future research.

## Conclusion

This study provides evidence that a history of concussion has effects on cortical processing and accuracy on a sensory grading task, and that these effects persist even after symptoms have resolved and individuals have returned to normal activities of daily living. Modulation of cortical responses based on stimulus relevance appears to be disrupted in people who have a history of concussion, which may contribute to differences in responding to task-relevant stimuli when faced with simultaneous distractors. As well, this study demonstrated a significantly greater task accuracy cost when distracting stimuli were presented to participants with a history of concussion than to controls. More research is required to characterize how concussion history affects the inter-related nature of top-down and bottom-up attentional orienting processes and to understand how electrophysiological and behavioral outcomes can be correlated to provide a more objective measure of recovery in this population.

## Data Availability Statement

The datasets generated for this study are available on request to the corresponding author.

## Ethics Statement

The studies involving human participants were reviewed and approved by Research Ethics Board, University of Waterloo. The patients/participants provided their written informed consent to participate in this study.

## Author Contributions

All authors contributed to the conception and design of the study. MA collected and analyzed the data and wrote the manuscript. All authors contributed to manuscript revision, read and approved the submitted version.

## Conflict of Interest

The authors declare that the research was conducted in the absence of any commercial or financial relationships that could be construed as a potential conflict of interest.
