# Supplementary figures and images for: A History of Concussion Affects Relevancy-Based Modulation of Cortical Responses to Tactile Stimuli
Source: Front Integr Neurosci. 2020 Jul 3;14:33. doi: 10.3389/fnint.2020.00033 (PMC7350857; doi:10.3389/fnint.2020.00033)

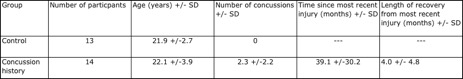

Supplement: Supplementary file 1 [file Image_1.JPEG]

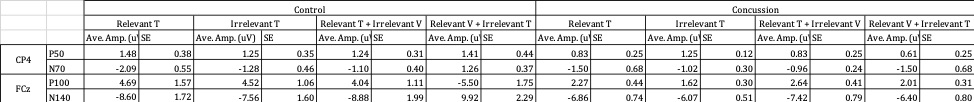

Supplement: Supplementary file 2 [file Image_2.JPEG]

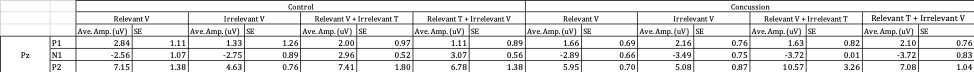

Supplement: Supplementary file 3 [file Image_3.JPEG]
